# Microbial Community Structure in the Rhizosphere of Rice Plants

**DOI:** 10.3389/fmicb.2015.01537

**Published:** 2016-01-13

**Authors:** Björn Breidenbach, Judith Pump, Marc G. Dumont

**Affiliations:** Biogeochemistry, Max Planck Institute for Terrestrial MicrobiologyMarburg, Germany

**Keywords:** *Oryza sativa*, rhizosphere, community ecology, bacteria, Archaea, *Herbaspirillum*, plant growth stage

## Abstract

The microbial community in the rhizosphere environment is critical for the health of land plants and the processing of soil organic matter. The objective of this study was to determine the extent to which rice plants shape the microbial community in rice field soil over the course of a growing season. Rice (*Oryza sativa*) was cultivated under greenhouse conditions in rice field soil from Vercelli, Italy and the microbial community in the rhizosphere of planted soil microcosms was characterized at four plant growth stages using quantitative PCR and 16S rRNA gene pyrotag analysis and compared to that of unplanted bulk soil. The abundances of 16S rRNA genes in the rice rhizosphere were on average twice that of unplanted bulk soil, indicating a stimulation of microbial growth in the rhizosphere. Soil environment type (i.e., rhizosphere versus bulk soil) had a greater effect on the community structure than did time (e.g., plant growth stage). Numerous phyla were affected by the presence of rice plants, but the strongest effects were observed for Gemmatimonadetes, Proteobacteria, and Verrucomicrobia. With respect to functional groups of microorganisms, potential iron reducers (e.g., *Geobacter, Anaeromyxobacter*) and fermenters (e.g., Clostridiaceae, Opitutaceae) were notably enriched in the rhizosphere environment. A *Herbaspirillum* species was always more abundant in the rhizosphere than bulk soil and was enriched in the rhizosphere during the early stage of plant growth.

## Introduction

Plants influence the spatial structure in soil by the growth of their roots (Angers and Caron, 1998). Further, they shape the chemical composition of the rhizosphere and provide microbial growth substrates by rhizodeposition (Lynch and Whipps, 1990; Hirsch et al., 2013; Philippot et al., 2013). Depending on the plant species, between 20 and 50% of plant photosynthate is transported belowground (reviewed by Kuzyakov and Domanski, 2000), and an average of 17% is released to the soil environment (reviewed by Jones et al., 2009). In return for growth substrates, rhizosphere microorganisms benefit plants by providing nutrients, phytohormones, suppressing phytopathogens or increasing resilience to abiotic stress such as heat, high salt, or drought (Welbaum et al., 2004; van der Heijden et al., 2008; Yang et al., 2009; Mendes et al., 2011; Nihorimbere et al., 2011; Haney et al., 2015). The microbial community is also largely responsible for the decomposition of organic matter in soil (Kuzyakov, 2002), which has consequences for biogeochemical cycling, soil formation, soil fertility and atmospheric trace gas formation (Conrad, 1996; Hinsinger et al., 2009).

Various factors shape the microbial communities associated with plants. A detailed study of *Arabidopsis thaliana* indicated that the endophytic microbial community has relatively low diversity compared with the bulk soil and is similar in plants cultivated in geochemically distinct soils (Lundberg et al., 2012). This indicates that the plant selects specific microorganisms to colonize its tissues. The *A. thaliana* rhizosphere microbial community also differed from the bulk soil, but soil geochemistry played a larger role in shaping the community. The same was true for maize; the microbial community of the rhizosphere was distinct from the bulk soil, but more similar than the rhizosphere communities of plants grown in different soils (Peiffer et al., 2013). Whereas soil geochemistry appears to be the primary determinant of the rhizosphere community structure, plant species (Marschner et al., 2004), cultivar (Micallef et al., 2009b; Inceoğlu et al., 2010; Edwards et al., 2015) and growth stage (Micallef et al., 2009a; Chaparro et al., 2014; Sugiyama et al., 2014; Sun et al., 2014) have all been shown to have significant additional effects.

Rice differs from most crops in that it is typically cultivated in flooded soil, resulting in oxic and anoxic zones within the rice rhizosphere that select for specific physiological groups of microorganisms with either aerobic, anaerobic, or facultivative metabolism (Brune et al., 2000). Methanogenesis in the rhizosphere and bulk soil of rice fields results in high methane (CH_4_) production, with rice agriculture currently contributing ∼10% of the global CH_4_ budget (Conrad, 2009). The primary substrates for methanogens are acetate or H_2_ + CO_2_ produced from the breakdown of complex carbon by the microbial community, including fermenters and acetogens (McInerney et al., 2008). Approximately 60% of CH_4_ produced in rice fields originates from root exudates or decaying root material (Watanabe et al., 1999).

The microbial communities inhabiting the rice field ecosystem have been described previously. For instance, the microbes within the rice root interior, the rhizoplane and the rhizosphere have been analyzed (Edwards et al., 2015). In addition, the microbial communities in various zones, such as rhizosphere, anoxic bulk soil, and oxic surface soil have been reported (Großkopf et al., 1998; Lüdemann et al., 2000; Lu et al., 2004; Asakawa and Kimura, 2008; Breidenbach and Conrad, 2015; Lee et al., 2015). Further studies have investigated the rice phyllosphere microbial community by 16S rRNA pyrotag sequencing (Ren et al., 2014) as well as endophytic and rhizospheric communities with metagenomic and metaproteomic approaches (Knief et al., 2012). Experiments have identified the bacteria (Lu et al., 2006; Hernández et al., 2015) and archaea (Lu and Conrad, 2005; Zhu et al., 2014) that consume plant-derived carbon in the rhizosphere. Also, specific functional groups of microorganisms, such as methanogens (Ramakrishnan et al., 2001; Lee et al., 2014) and methanotrophs (Henckel et al., 1999; Eller and Frenzel, 2001; Ho et al., 2011; Lee et al., 2014), have been extensively analyzed in rice systems. However, none of these studies focused on the impact of the rice plant on the total microbial community and over several growth stages of rice.

The objective of this study was to determine the extent to which rice plants influence the microbial community in a rice field soil. Secondly, we aimed to determine if plant growth stage had an effect on the microbial community composition in the rhizosphere. The experiment was performed under greenhouse conditions to minimize confounding factors, and using a soil from Vercelli (Italy) with a long history of rice cultivation to avoid changes that could result in a soil that was not adapted to flooding or the growth of rice plants. Similarly, it was necessary to use the rice variety cultivated in the Vercelli rice fields, otherwise there could have been changes to the microbial community as a result of adaptation to an unfamiliar plant genotype (Edwards et al., 2015). The first sampling was past the initial dynamic phase after flooding in order to focus on the influence of the plant and not the effects of flooding. The analysis provides information on the difference between the rice rhizosphere and bulk soil microbial community across different plant growth stages showing how the rice plant shapes the microbial community composition in a mature rice field soil.

## Materials and Methods

### Microcosms and Incubations

Soil was sampled from rice fields at the Italian Rice Research Institute in Vercelli, air-dried and stored at room temperature until the start of the experiment. A detailed analysis of the physiochemical properties of the soil has been published previously (Pump and Conrad, 2014). Immediately prior to the establishment of microcosms, soil was sieved through a stainless steel screen (0.2 mm mesh) and 2.5 kg was added to opaque plastic pots (16 cm height, 17.5 cm diameter). The pots were flooded with deionized water 1 week before planting. Fertilizers included urea (CH_4_N_2_O, 45 g l^-1^) as nitrogen source, phosphorus (Na_2_HPO_4_⋅2H_2_O, 17 g l^-1^), potassium (KCl, 50 g l^-1^), and magnesium (MgSO_4_⋅7H_2_O, 2 g l^-1^). The phosphorus, potassium, and magnesium solutions were added at a ratio of 10 ml kg^-1^ soil 1 day before planting, whereas 5 ml kg^-1^ of the urea was added twice, 1 day before planting and after 14 days of plant growth. Rice seeds (*Oryza sativa* var. Koral) were also obtained from the Rice Research Institute in Vercelli, Italy. The rice seeds were treated with the fungicide Aatiram and germinated at 25°C and 75% humidity in a greenhouse. Three germinated rice seedlings were planted each in a total of 20 pots. A further five pots were left unplanted. The pots were incubated in a greenhouse at 25°C and 75% humidity with a 12 h light/dark cycle. Pots were watered daily to maintain approximately 3 cm water overlying the soil. Plant heights and tiller number were recorded weekly. Five planted pots were sacrificed after 34, 52, 62, and 90 days after planting (stages 1–4) and rhizosphere soil (planted pots), bulk soil (unplanted pots) and pore water were collected aseptically using sterilized equipment. Plants were extracted from the pots and shaken to remove large soil aggregates and adhering soil. The soil remaining attached on the roots was considered to be rhizosphere soil and was sampled using a sterile spatula. Samples were immediately frozen in liquid nitrogen and stored at -80°C until further analysis. The water content of each soil was determined gravimetrically by drying subsamples at 60°C until they reached a constant weight.

### Porewater Analysis

Soil samples (100 g) were centrifuged for 10 min at 20,000 *g* and 4°C in 50-ml centrifuge tubes. The supernatant was filter sterilized using 0.2 μm acetate-free filters (GE Healthcare Life Science, Freiburg, Germany) and stored at -20°C until analysis. Organic acids were analyzed with high performance liquid chromatography [HPLC; pump S1000, oven S4110 (Sykam, Germany), sampler Jasco 851-AS Intelligent Sampler (JapanSpectroscopy Co. Ltd., Japan) with an Aminex HPX-87 H organic acid column (Bio-Rad, Germany)]. Technical specifications were as follows: 65°C oven temperature, 2 mM H_2_SO_4_ as eluent, 0.5 ml/min flow rate and a UV detector at 40°C. Inorganic ions including chloride, nitrate, nitrite, phosphate, and sulfate were detected using ion chromatography [IC; pump S1121, sampler S5200 (Sykam); Bak et al., 1991]. Concentrations of organic acids and inorganic ions between the treatments were compared using analysis of variance (ANOVA) followed by Tukey’s *post hoc* test in R version 3.02 (R Core Team, 2013).

### Nucleic Acid Extraction

Soil DNA was extracted using the NucleoSpin^®^ Soil Kit (Macherey-Nagel, Düren, Germany) following the manufacturer’s instructions. DNA concentration and purity were determined spectrophotometrically (NanoDrop Technologies, USA). All DNA samples had absorbance ratios of A260:A230 > 1.7 and A260:A280 > 1.8.

### Real-Time Quantitative PCR

Quantitative PCR (qPCR) was used to quantify bacterial and archaeal 16S rRNA gene copies using primers Ba519f/Ba907r (Stubner, 2002) and Ar364f/Ar934br (Burggraf et al., 1997; Großkopf et al., 1998) respectively. All reactions were set-up on ice with minimal light exposure. Each reaction included the following: 4 mM MgCl_2_, 0.01 μM fluorescein calibration dye (Bio-Rad), 0.625 U Jump Start^TM^ TaqReadyMix^TM^, 0.60 μM of each primer, 1 μL template DNA. Standards containing known numbers of DNA copies of the target gene were serially diluted to serve as calibration curves. The qPCRs were performed on an iCycler thermocycler equipped with a MyiQ detection system (Bio-Rad, Munich, Germany). The following program was used for amplification of archaeal 16S rRNA gene copies: 94°C for 6 min, followed by 40 cycles of 94°C for 35 s, 66°C for 30 s and 72°C for 45 s. Fluorescence was measured after each cycle at a temperature of 86.5°C. A melting curve was performed from 75 to 95°C. The program for amplification of bacterial 16S rRNA gene copies was as follows: 94°C for 8 min, followed by 50 cycles of 94°C for 20 s, 50°C for 20 s, and 72°C for 50 s. A melting curve was performed from 75 to 95°C. Data analysis was performed using Bio-Rad IQ5 2.0 Standard Edition Optical System Software (Bio-Rad). A Kolmogoroff–Smirnoff test indicated that the qPCR data was normally distributed. Means of copy numbers between the treatments were compared using ANOVA followed by Tukey’s *post hoc* test in R version 3.0.2 (R Core Team, 2013).

### 16S rRNA Amplicon Pyrosequencing and Sequence Analysis

A total of 24 samples were chosen for amplicon pyrosequencing. Samples corresponded to three replicate microcosms chosen at random from each of the two treatments (rhizosphere, bulk soil) and the four sampling time points. The 16S rRNA genes of bacteria and archaea were targeted with primers F515 and R806 described previously (Bates et al., 2011). The forward primer was tagged with 6-base barcodes. Sequencing of the PCR products was performed at the Max Planck Genome Centre in Cologne using a Roche 454 Genome Sequencer GS FLX+. Data are available in the NCBI sequence read archive (SRA) with the accession number SRP057189.

Sequences were quality filtered and grouped into OTUs (97% identity) with the UPARSE pipeline (Edgar, 2013). A maximum expected error threshold of 0.5 was used and sequences were trimmed to 200 bp. Sequences were excluded if they contained mismatches to the forward primer, were shorter than 200 bp or contained ambiguities. Chimeras were removed using the UCHIME *de novo* algorithm. Representative sequences of OTUs were classified using the silva taxonomy and the Wang (naïve Bayesian classifier) method implemented in *mothur* version 1.31.2 (Schloss et al., 2009). Relative abundances of OTUs between samples were analyzed using the *vegan* package version 2.2-1 (Oksanen et al., 2013) in R version 3.0.2 (R Core Team, 2013).

### Statistical Analyses and Data Exploration

Statistical analyses were performed using R version 3.0.2 (R Core Team, 2013). ANOVA and principal components analysis (PCA) were performed using package *stats* (R Core Team, 2013). Data was tested for normality before performing ANOVA. Linear regression models were calculated using the function *lm* within *stats*. Constrained correspondence analysis (CCA) was performed using the function *cca* in the *vegan* package (Oksanen et al., 2013). PCA was performed using the *prcomp* function of Hellinger distances (i.e., Euclidean distances of Hellinger transformed data) and the 50 OTUs contributing the largest absolute loadings in the first dimension were obtained from the rotation output file. A heatmap was prepared using the *gplots* package (Warnes et al., 2015); the samples were clustered with the *hclust* function using the ‘Ward’ method based on various distances/dissimilarities (Euclidean, Manhattan, Bray–Curtis) calculated with the *vegdist* function in the *vegan* package. Diversity analyses (Shannon index, coverage) were performed using the diversity calculators within the *vegan* package; the dataset was first randomly subsampled to 3699 sequences per sample using the *rrarefy* function in *vegan*. Differences in population structure were tested by ANOSIM (Clarke, 1993) based on Bray–Curtis dissimilarities within the *vegan* package. Differences between sites (rhizosphere versus bulk soil) or differences based on sampling time were examined.

## Results

### Characterization of Soils in Microcosms

The determination of the growth stage was conducted by monitoring rice plant height and tiller number (Supplementary Figure S1). Four sampling time points were selected corresponding to stage 1 (day 34, early vegetative), stage 2 (day 52, late vegetative), stage 3 (day 62, reproductive), and stage 4 (day 90, maturity). Soil pore water analyses indicated that lactate, formate, acetate, chloride, and propionate concentrations were higher in the planted pots, but these differences were only statistically significant for chloride and propionate (**Table 1**). The concentrations of malate, nitrate, and sulfate were similar between rhizosphere (planted pots) and bulk soil (unplanted pots).

**Table 1:** Concentrations of organic acids and inorganic ions detected in the pore water of rhizosphere (planted) and bulk soil (unplanted) at stages 1–4, corresponding to days 34, 52, 62, and 90.

	Planted [days]				Unplanted [days]			
	34	52	62	90	34	52	62	90
Malate	70.23a ± 23.05	69.18a ± 25.17	103.51a ± 11.45	121.92a ± 19.63	85.96a ± 9.43	101.31a ± 8.37	119.89a ± 58.70	148.13a ± 88.17
Lactate	0.98a ± 1.01	1.41a ± 1.39	1.16a ± 0.40	1.26a ± 0.53	0.17a ± 0.18	0.01a ± 0.02	0.03a ± 0.07	0.04a ± 0.07
Formate	0.13a ± 0.14	0.10a ± 0.10	0.31a ± 0.14	0.40a ± 0.09	0.03a ± 0.05	0.09a ± 0.02	0.05a ± 0.03	0.04a ± 0.03
Acetate	0.44a ± 0.42	0.50a ± 0.56	0.48a ± 0.41	0.55a ± 0.41	0.02a ± 0.04	0.01a ± 0.01	ND	0.01a ± 0.01
Propionate	0.06 ± 0.09	1.17 ± 1.69	0.38 ± 0.20	0.96 ± 0.35	ND	ND	0.03 ± 0.00	ND
Chloride	12.70a ± 4.08	30.95bc ± 2.40	29.06bc ± 5.98	34.91b ± 2.08	12.05a ± 1.07	14.49a ± 1.64	16.48a ± 3.36	19.10ac ± 4.52
Nitrate	0.02a ± 0.02	0.01a ± 0.01	0.05a ± 0.05	ND	0.13a ± 0.20	0.05a ± 0.05	0.14a ± 0.15	0.23a ± 0.30
Sulfate	0.03a ± 0.03	0.02a ± 0.02	0.02a ± 0.01	0.03a ± 0.02	0.02a ± 0.02	0.02a ± 0.02	0.03a ± 0.02	0.05a ± 0.03

*Values indicate the average and standard deviation in millimolar concentrations. Values with the same letters are not different (ANOVA, P threshold 0.05).*

*ND indicates not detected.*

### Bacterial and Archaeal 16S rRNA Abundance

**FIGURE 1:**
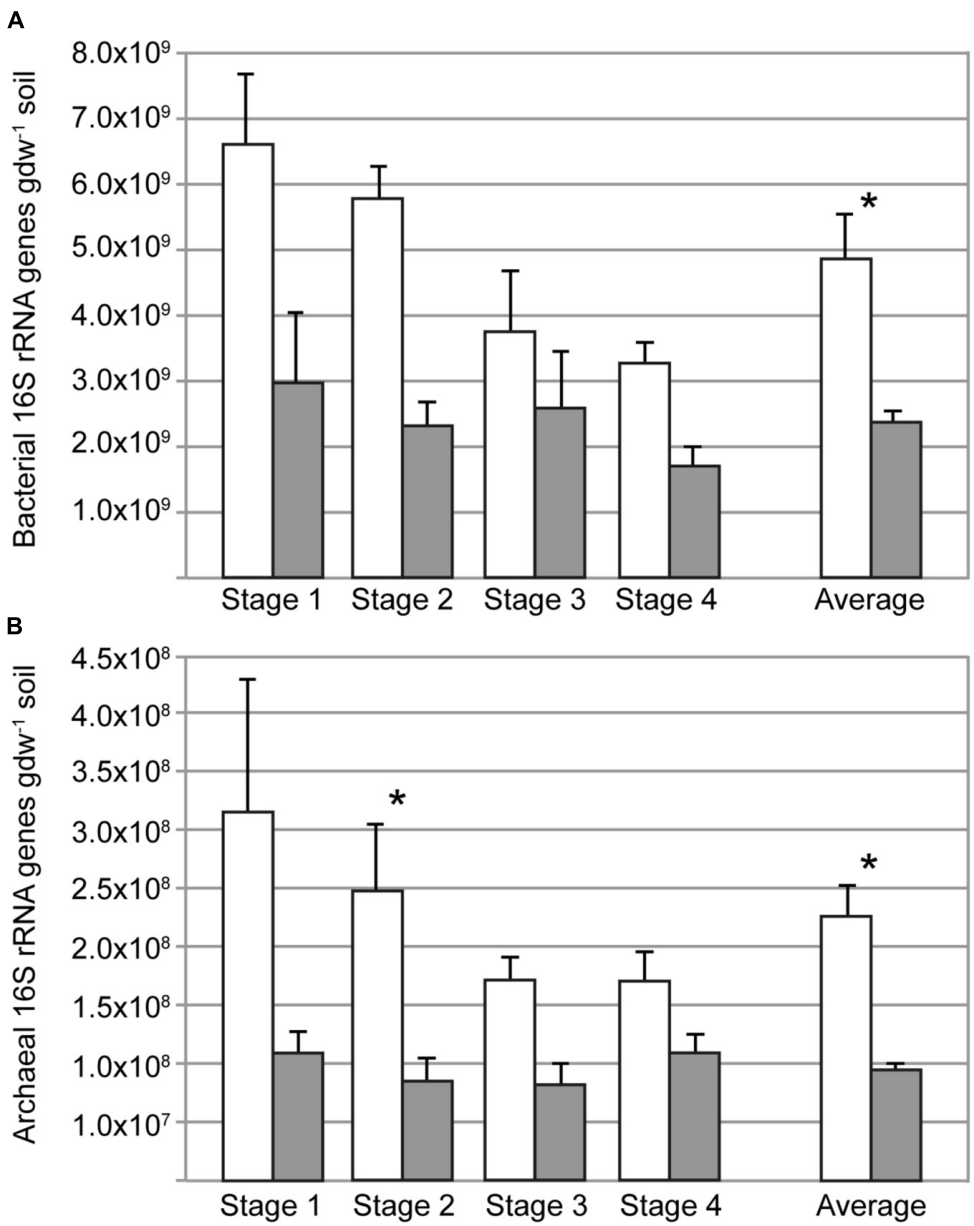
**Copy numbers of bacterial (A) and archaeal (B) 16S rRNA genes in rhizosphere (open bars) and bulk soil (filled bars) at different sampling times (days 34, 52, 62, 90); the average of the four sampling times is also shown.** Asterisks indicate when the abundance in rhizosphere and bulk soil was different (ANOVA, *P* < 0.05). Error bars correspond to standard errors of means from replicate soil samples (*n* = 5).

The influence of the rice plant on the microbial community size was determined by qPCR assays targeting the bacterial and archaeal 16S rRNA gene. Both bacterial and archaeal 16S rRNA abundances were about twofold higher in rhizosphere (planted pots) than in soil from bulk soil (unplanted pots; **Figure 1**). Temporal changes were not detected in the bulk soil. The abundance of bacterial and archaeal 16S rRNA gene copies decreased from stages 1 to 4, but this was not statistically significant (ANOVA, *P* > 0.05). The 16S rRNA gene copy numbers of bacteria were approximately 20 times larger than those of archaea.

### Bacterial and Archaeal Diversity

Pyrosequencing of the bacterial 16S rRNA gene was performed to characterize the microbial communities. After processing and chimera removal, an average of 7300 sequences (±2500) were obtained for each sample. The sequences grouped into 8685 OTUs at 97% identity. Coverage was between 91 and 95% for each of the eight sample types (bulk soil and rhizosphere at four sampling times). Diversity indices were neither different between rhizosphere and bulk soils nor between sampling times.

**FIGURE 2:**
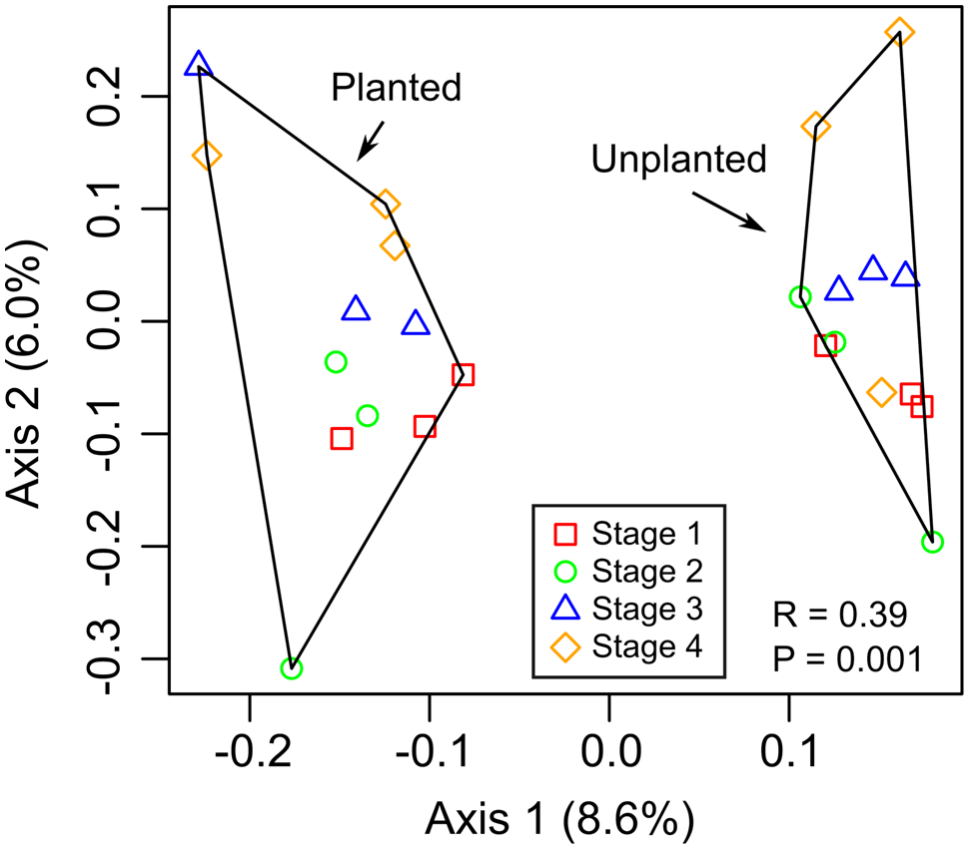
**Principal component analysis (PCA) based on relative abundance of 16S rRNA gene OTUs.** The ANOSIM statistic was applied to the ordinations to test for differences between rhizosphere (planted) and bulk (unplanted) soil communities, with corresponding *R-* and *P*-values shown.

The microbial communities separated by rhizosphere and bulk soil based on PCA of OTUs (97% 16S rRNA identity), indicating that there was a difference in the community composition (ANOSIM, *R* = 0.39, *P* = 0.001; **Figure 2**). Despite this clear separation, the first axis only explained 8.6% of the variance in the data, indicating that the relative abundance of most OTUs were similar between rhizosphere and bulk soils. There was no apparent clustering of rhizosphere samples based on plant growth stage or the corresponding sampling time in unplanted (bulk) soil microcosms. Sequences were classified and summarized by bacterial phylum, which did not show differences (ANOVA, *P* > 0.05) between rhizosphere and bulk soil samples (Supplementary Figure S2), indicating the overall composition of the community at low phylogenetic resolution did not change between these environments. CCA was performed to test the extent to which pore water data (**Table 1**) explained the community composition (Supplementary Figure S3). Only chloride was significant.

**FIGURE 3:**
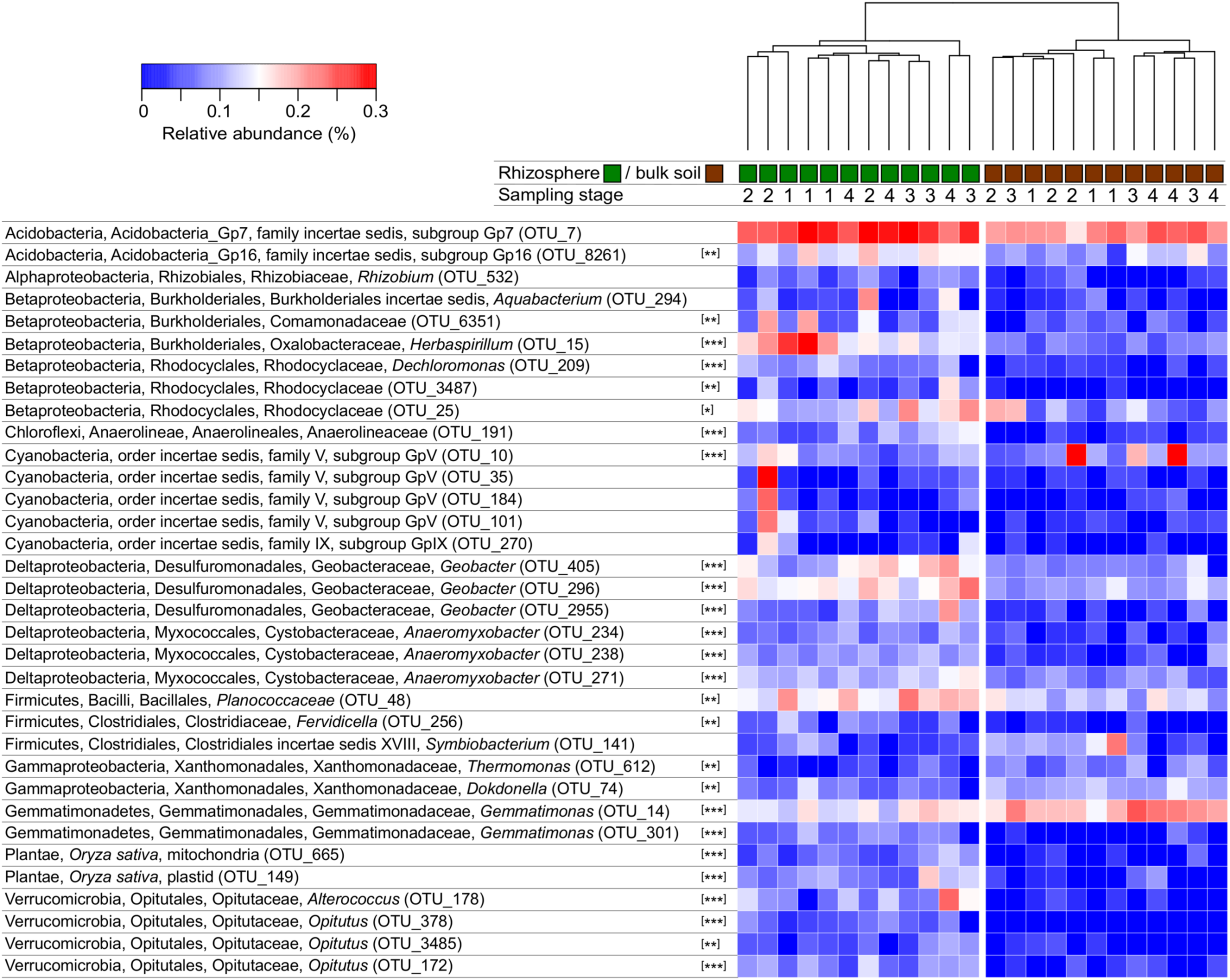
**Heatmap depicting the relative abundance of OTUs with greatest dissimilarity between rhizosphere and bulk soils.** Clustering of samples is shown based on Manhattan distances and was similar using Bray–Curtis dissimilarity. OTUs were classified and only those that could be classified to order or lower were included. The sampling times (1–4) correspond to 34, 52, 62, and 90 days after planting. Asterisks indicate when the abundance of an OTU was different in rhizosphere and bulk soil (ANOVA, ^∗^*P* < 0.05, ^∗∗^*P* < 0.01, ^∗∗∗^*P* < 0.001).

A heatmap was constructed to depict the relative abundance of OTUs that best represent the dissimilarity between the microbial communities in rhizosphere versus bulk soil by selecting 50 with the largest loadings of the PCA. Among these 50 OTUs, 34 could be classified to family level or lower and are depicted in a heatmap (**Figure 3**); data for all 50 OTUs are provided in the supplement. The rhizosphere and bulk soil samples clustered independently with each of the distance/dissimilarity indices tested. ANOVA was used to test if their relative abundance between rhizosphere and bulk soil was significantly different. Most of the OTUs were significant, with some exceptions including several Cyanobacteria that were abundant in one rhizosphere sample in stage 2 (**Figure 3**). The rhizosphere soil contained a higher abundance of rice mitochondria and plastid 16S rRNA sequences. This was anticipated since root cells are sloughed-off into the rhizosphere, and therefore provides a confirmation of the ability of the analysis to detect differences between the rhizosphere and bulk soil communities. The Shannon diversity index for these 50 OTUs in rhizosphere was 3.08 ± 0.14 and 2.55 ± 0.25 for the bulk soil. Among these 50 OTUs, 42 had higher average relative abundance in rhizosphere than bulk soil and eight had higher abundance in bulk than rhizosphere soil (Supplementary data); given that the number of 16S rRNA genes was twofold larger in the rhizosphere than bulk soil, it is possible that even more than 42 of the OTUs were higher in rhizosphere soil based on absolute abundance.

**FIGURE 4:**
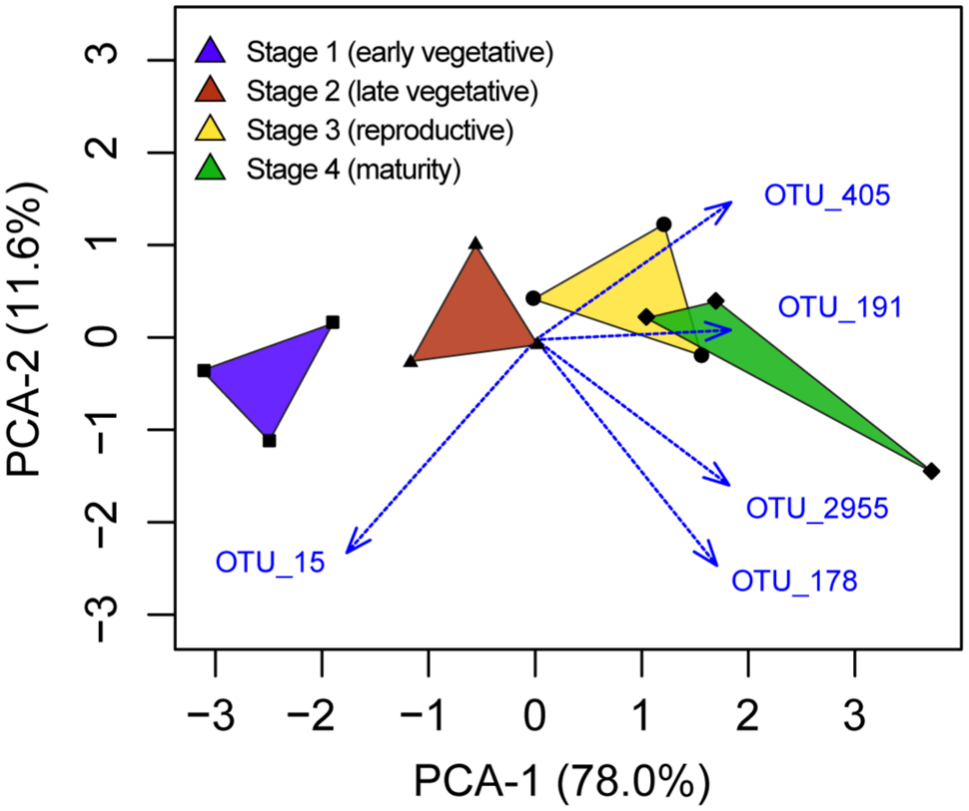
**Principal component analysis depiction of the five OTUs identified to have different relative abundances between plant growth stages**.

Among the 42 OTUs that were enriched in rhizosphere soil, we aimed to determine if some were selected at a specific plant growth stage. The five OTUs with higher relative abundance in one stage compared with any other stage (ANOVA, *P* < 0.05) are depicted in a PCA analysis (**Figure 4**). OTU_15, corresponding to an uncultivated *Herbaspirillum* species, was the only OTU enriched in stage 1. There was a difference in relative abundance for OTU_15 between stages 1 and 4 (Tukey’s *post hoc* test). The other four OTUs were more abundant in stages 3 or 4 compared with stage 1. These corresponded to two *Geobacter* species (OTU_405, OTU_2955), one Anaerolineaceae (OTU_191) and one *Alterococcus* (OTU_178).

**FIGURE 5:**
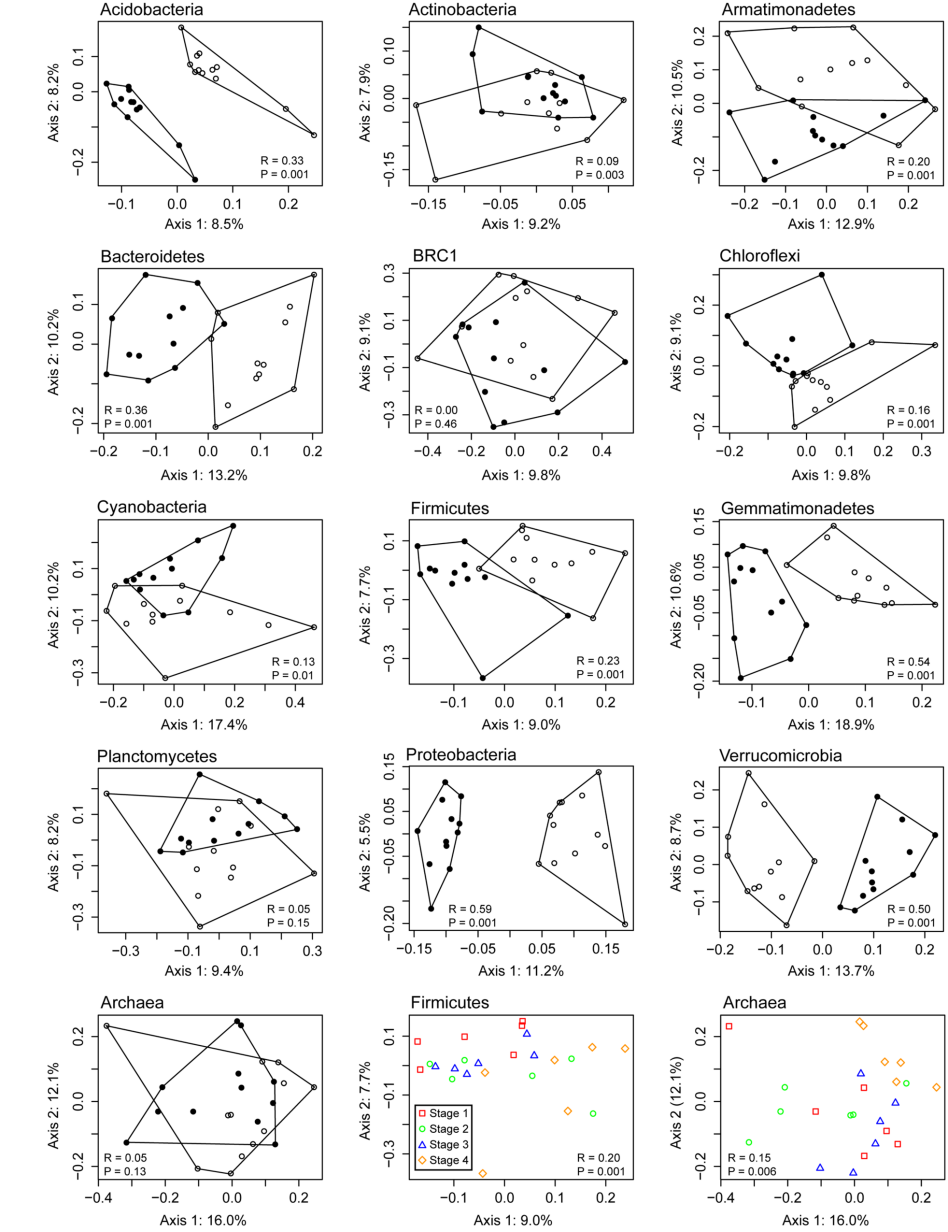
**Principal coordinates analysis (PCoA) of 16S rRNA gene OTUs assigned to major microbial phyla detected in Vercelli rice field soil.** Bulk soil samples are depicted with filled symbols and rhizosphere samples with open symbols. The ANOSIM statistic was applied to the ordinations to test for differences between rhizosphere and bulk soil communities, with corresponding *R-* and *P*-values shown; the last two plots show the *R-* and *P*-values based on differences between sampling times for Firmicutes and Archaea respectively.

Although the PCA/heatmap provided hints as to which OTUs were differentially abundant between rhizosphere and bulk soil microcosms, we wanted to determine if this pattern could be seen for individual phyla alone. To determine this, OTUs were classified and separated into phylum-level groups, which were then analyzed individually by principal coordinates analysis (PCoA; **Figure 5**). When testing for differences between rhizosphere and bulk soil communities, the ANOSIM statistic was largest (*R* ≥ 0.5) for Gemmatimonadetes, Proteobacteria and Verrucomicrobia, indicating clear differences in community structure for these taxa. No difference was detected (*R* = 0) for the candidate phylum BRC1, or differences were not significant (*P* > 0.05) for Planctomycetes and Archaea. Differences in population structures based on growth stages were only significant for Firmicutes and Archaea, but in both cases there was overlap between the stages (**Figure 5**). Separate analyses of Bacillales and Clostridiales within the Firmicutes showed a difference in communities between bulk and rhizosphere soils for both classes, but the temporal pattern was only significant for Clostridiales (*R* = 0.18, *P* = 0.001). For Archaea, one cause of the temporal pattern was a higher relative abudance of *Methanosarcina* and *Methanosaeta* in stage 4 compared with the earlier sampling times, although this difference was not statistically significant. Linear models were used to test for correlations between *Methanosarcina* and *Methanosaeta* relative abundances with pore water data (**Table 1**). Among these, the only significant relationship was a positive correlation between *Methanosarcina* and formate concentration.

## Discussion

The rhizosphere is known as a compartment in the soil influenced by the plant and where organic matter is introduced via rhizodeposition and sloughed-off cells. Rhizodeposition by rice plants has been described (Aulakh et al., 2001; Wu et al., 2009) and shown to enhance microbial activity in the rhizosphere compared with bulk soil (Butler et al., 2003). Oxygen is also released from rice roots (Frenzel et al., 1992; Colmer and Pedersen, 2008) and serves as an electron acceptor for aerobic microorganisms and is likely to influence the microbial populations and the chemical transformations that occur. The volume and composition of organic molecules released from roots changes somewhat with rice plant growth stage (Aulakh et al., 2001), which in theory could also cause temporal shifts in the rhizosphere microbial community. Although not statistically significant, our data (**Table 1**) supports other studies that show the combined influence of the rice plant and the microorganisms in the rhizosphere act to modify the chemical composition of the soil environment. Most importantly, in this study we show the extent to which these changes occur in the rice rhizosphere and the degree to which growth stage influences the microbial community.

There were relatively few temporal changes in the microbial community either in bulk or rhizosphere soil. This is consistent with a previous study (Noll et al., 2005) of Vercelli rice field soil where changes in the bacterial community were observed only during the first 21 days after flooding. This initial phase is dominated by a succession of r-strategists that presumably consume readily available growth substrates, energy sources and high-redox potential electron acceptors (Noll et al., 2005; Shrestha et al., 2007). Interestingly in that study, a difference between the surface and subsurface soil was also not observed beyond day 21 (Noll et al., 2005). Another study also examined changes in a time-series within the first 2 weeks after planting (Edwards et al., 2015). Our first sampling time was 34 days after planting, equivalent to 41 days after flooding, and therefore well-beyond this initial dynamic phase. This was important for our experimental design as we could focus on the influence of the plant and not the effects of flooding.

Previous reports have shown that the difference in microbial communities between the bulk and rhizosphere soil of land plants is less than the difference between soils with different locations or geochemistry (Lundberg et al., 2012; Peiffer et al., 2013; Edwards et al., 2015). A study of rice field showed that the microbial communities clustered by soil depths, which affects oxygenation and redox potential, rather than by bulk and rhizosphere soil (Lee et al., 2015). We sampled only the soil adhered to the rice root or unplanted bulk soil below the oxygen diffusion zone, therefore, making the comparison of rhizosphere and bulk soil communities more straightforward. Although there was a doubling of the bacterial community in the rhizosphere as determined by qPCR targeting bacteria 16S rRNA genes, there was no significant difference in the relative abundance at the phylum level. This indicates that the changes in relative abundance was not restricted to a small number of taxa within a limited number of phyla, otherwise the relative abundance of the stimulated phyla would have increased and unresponsive phyla would have decreased relative to the bulk soil. This was confirmed by the separate analysis of phylum-level microbial populations, which showed that with the exception of candidate division BRC1, Planctomycetes and Archaea, all the community structures of individual phyla were distinct between rhizosphere and bulk soil. Therefore, the rice plant restructured nearly all the phylum-level microbial groups, which can explain the doubling of bacterial abundance in the rhizosphere soil.

The absolute abundance of Archaea, like Bacteria, was on average more than twofold greater in rhizosphere than bulk soil, but the archaeal community composition based on relative abundance of OTUs was not significantly shaped by these locations. As discussed above, an increase in the absolute abundance of a group without a change in the relative abundance of the individual OTUs would indicate an equal stimulation of all taxa, which appeared to be the case of Archaea in our experimental system. Studies have shown that archaeal communities are relatively stable once established and are resistant to environmental perturbation, such as drainage (Krüger et al., 2005; Watanabe et al., 2006; Scavino Fernandez et al., 2013; Breidenbach and Conrad, 2015), or oxygenation in the case of strict anaerobes (Yuan et al., 2009). Methanogens such as Methanosarcinaceae and Methanocellaceae possess various genes encoding for oxygen detoxifying enzymes (Erkel et al., 2006; Angel et al., 2011) thus probably allowing them to survive exposure to oxygen in the rhizosphere. Another study has proposed Methanosaetaceae to tolerate oxygen better than previously believed (Brandt et al., 2015). The lower abundance of Archaea in the bulk soil compared with rhizosphere might be a reflection of energy or carbon limitation in the absence of rice plants. Our current hypothesis is that the relative abundance of Archaea in Vercelli rice field soil is shaped by their growth in the rhizosphere, and that archaeal OTUs respond proportionally to carbon or energy limitation. Whereas the community is relatively stable, their activity is likely to fluctuate. For example, studies have shown that *mcrA* gene expression in methanogens varies during rice plant development (Lee et al., 2014), after exposure to oxygen (Yuan et al., 2009) or at different times of the day in the rice rhizosphere (Xu et al., 2012), but under most of these conditions there was no change in methanogen numbers.

We also identified specific OTUs that had different relative abundance between rhizosphere and bulk soil. Three OTUs corresponding to each of *Geobacter* and *Anaeromyxobacter* were significantly more abundant in the rhizosphere. Both these genera have been shown to be important iron reducers in Vercelli soil (Hori et al., 2010). Hori et al. (2010) specifically showed that they use acetate as a carbon source and oxidized iron, in the form of either ferrihydrite or goethite, as an electron acceptor. They also identified uncultivated Rhodocyclaceae that grew in the presence of goethite, which might also explain the higher abundance of Rhodocyclaceae in rhizosphere soil in our experiment. Iron reduction can be sustained in the rice rhizosphere by the exudation of acetate by roots and the chemical or biological oxidation of reduced iron (Conrad and Frenzel, 2002). The rhizosphere also contained higher relative abundances of *Opitutus* and *Clostridia* OTUs. These are well known as anaerobes with fermentative metabolism (Andreesen and Schaupp, 1973; Chin et al., 2001) and OTUs belonging to both these genera were identified consumers of plant-derived carbon in the rice rhizosphere of Vercelli soil (Hernández et al., 2015).

Compared with the difference between bulk and rhizosphere soil, there was only a relatively minor effect of plant growth stage on the microbial community composition. The ANOSIM analysis showed significant temporal patterns for Firmicutes and Archaea only, and both demonstrated high overlap between the sampling stages. This temporal pattern of Firmicutes was restricted to Clostridiales, which are known fermenters of plant material in flooded soil (Rui et al., 2009). Previous studies in rice field soil has shown them to be among the first to proliferate after flooding (Noll et al., 2005; Rui et al., 2009). The difference in the composition of Firmicutes between bulk and rhizosphere soil was still slightly stronger than the temporal differences. In contrast, we observed a difference in archaeal community composition only with time and not between bulk and rhizosphere soils. In other words, although the rice plants caused a doubling of the Archaea in the rhizosphere compared with the bulk soil, their community composition was influenced more by time after flooding than by the rice plant. Previous studies of methanogens, which are the dominant Archaea in Vercelli soil, have shown their activity to change with time after flooding (Yao and Conrad, 1999; Yao et al., 1999). This temporal effect is controlled or influenced by a variety of factors, including the presence of high potential inorganic electron acceptors and the availability of degradable organic substrates (Conrad and Frenzel, 2002).

We were interested in identifying OTUs enriched in the rhizosphere at one of the plant growth stages. OTU_15, corresponding to a *Herbaspirillum* species, was the only OTU identified as enriched in stage 1. Many *Herbaspirillum* have been identified as endophytic plant growth promoting bacteria (Elbeltagy et al., 2001; Rothballer et al., 2008). Baldani et al. (2000) identified *Herbaspirillum seropedicae* strains that could provide up to 54% of rice seedling nitrogen. Another study showed that phosphate-solubilizing *Herbaspirillum* strains could provide further rice plant growth enhancement under certain conditions (Estrada et al., 2013) and may have other plant growth promoting mechanisms. More research is needed to determine how beneficial strains of *Herbaspirillum* could be enriched within the rhizosphere and endosphere early during plant development and how this might be used to decrease fertilization requirements.

Finally, there were four OTUs identified as being significantly enriched in the late growth stage 4. Two of the OTUs corresponded to *Geobacter*. As discussed above, *Geobacter* are known iron reducers that consume plant carbon in the rice rhizosphere (Hori et al., 2010; Hernández et al., 2015). Their activity is of particular importance since it consumes organic substrates that could otherwise be converted to CH_4_, and studies have shown that the addition of oxidized iron decreases methanogenesis in rice field soil (Achtnich et al., 1995; Chidthaisong and Conrad, 2000; Jäckel et al., 2005). CH_4_ released to the atmosphere from rice fields is a global concern (Bodelier, 2015), and understanding how the abundance and activity of *Geobacter* can be enhanced in the rice rhizosphere might lead to strategies for decreasing methanogenesis.

## Conclusion

This study showed that rice plants have a extensive effect on the microbial community composition, which was detectable across all bacterial phyla except BRC-1 and Planctomycetes. The majority of the OTUs with a significant response had higher relative abundance in the rhizosphere compared with bulk soil. Although the differences in relative anbundance of OTUs between bulk and rhizosphere were moderate (maximum ∼5-fold), it shows how the rice plant shapes and maintains the microbial community composition in rice field soil. The archaeal community composition did not change between bulk soil and rhizosphere despite an increased abundance in the latter, suggesting that their community composition is relatively stable in Vercelli rice field soil. Future studies should focus on understanding how specific microbial species, such as *Herbaspirillum* and *Geobacter*, are enriched in the rhizosphere at different growth stages and whether this involves plant signaling mechanisms (Hassan and Mathesius, 2012). Strategies to enhance the activity of these microorganisms might decrease fertilization requirements or lead to a mitigation in greenhouse gas formation, both of which are priorities for rice agriculture.

## Author Contributions

BB designed and performed experiments, analyzed the data, and wrote the manuscript. JP provided reagents and designed and performed experiments. MD designed the experiments, analyzed the data, and wrote the manuscript.

## Conflict of Interest Statement

The authors declare that the research was conducted in the absence of any commercial or financial relationships that could be construed as a potential conflict of interest.
