# Supplementary material for: Microbial Community Structure in the Rhizosphere of Rice Plants
Source: Front Microbiol. 2016 Jan 13;6:1537. doi: 10.3389/fmicb.2015.01537 (PMC4710755; doi:10.3389/fmicb.2015.01537)
Supplement: Supplementary file 1 [file Supplementary_Figures.PDF]

## Supplementary figures

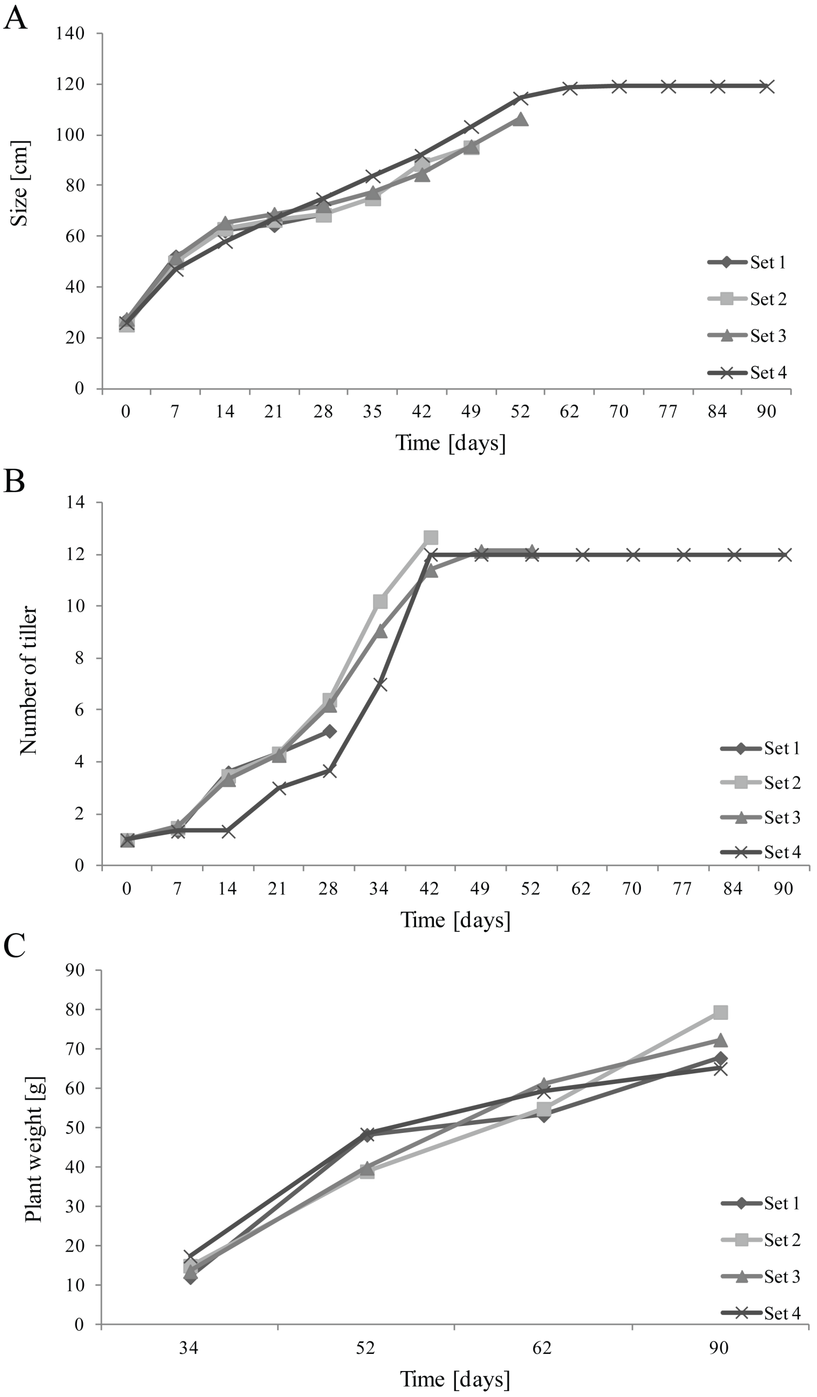


**Figure S1.** Basic characteristics of plant growth.

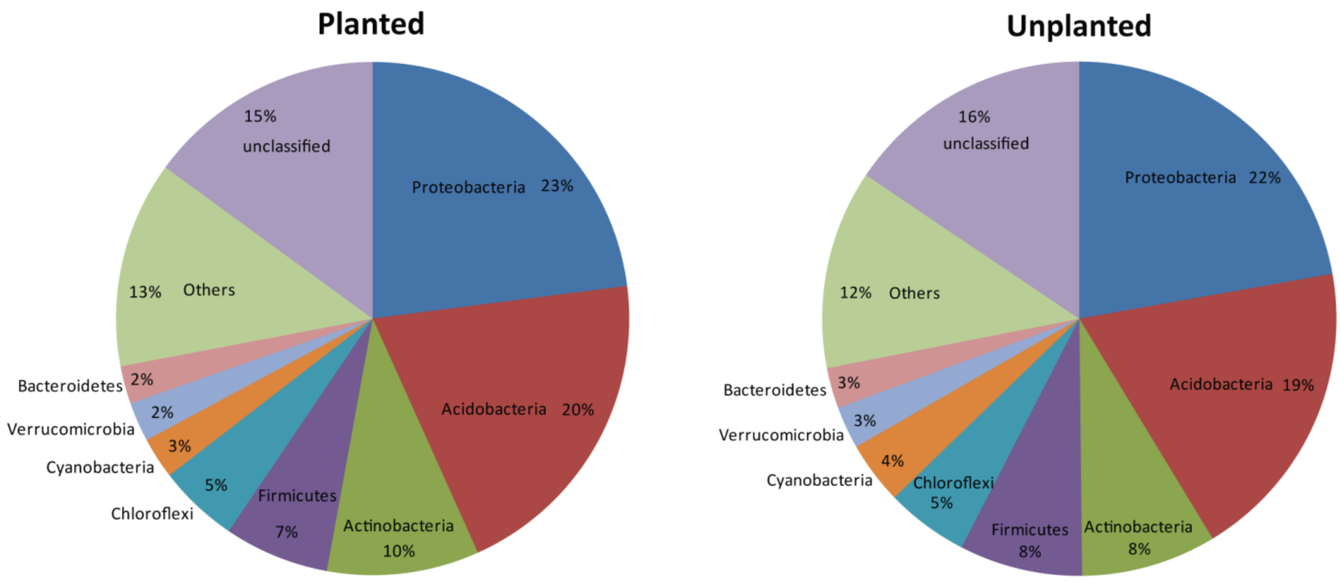


**Figure S2.** Phylum level comparison of bacteria abundance in rhizosphere (planted) and bulk soil (unplanted) based on 454 pyrosequencing analysis of 16S rRNA genes. All phyla contributing less than 2% of sequences were summarized as others.

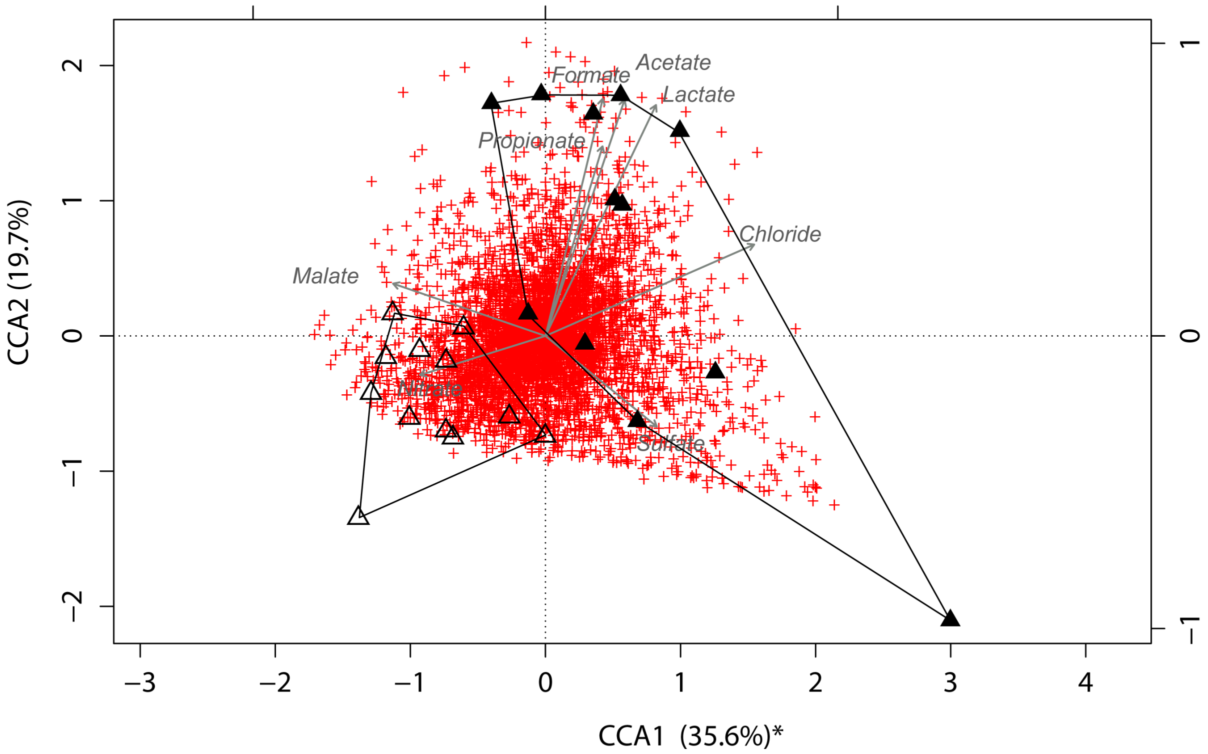


**Figure S3.** Canonical correspondence analysis of OTU abundances constrained by organic and inorganic ion data. OTUs are represented by red squares and samples are represented by triangles (open are rhizosphere and filled are bulk soil samples).
